# Stroke services in MENA: What is there and what is needed

**DOI:** 10.1371/journal.pone.0288030

**Published:** 2023-07-20

**Authors:** Hany Aref, Nevine El Nahas, Suhail Abdulla Alrukn, Maria Khan, Selma Kesraoui, Firas Alnidawi, Elyar Sadeghi Hokmabadi, Mehdi Farhoudi, Muataz Fairooz, Waleed Batayha, Athari Salmeen, Husen Abdulrahman, Mohammed Faouzi Belahsen, Amal M. Al Hashmi, Naveed Akhtar, Mohammed Al-Johani, Osheik Abu’Asha Seidi, Anas Jouhar, Chokri Mhiri, Ahmed Elbassiouny, Hossam Shokri, Tamer Roushdy

**Affiliations:** 1 Neurology Department, Faculty of Medicine, Ain Shams University, Cairo, Egypt; 2 Neurology Department, Rashid Hospital, Dubai, United Arab Emirates; 3 Neurology Department, Blida Hospital University, Blida, Algeria; 4 Neurology Department, Salmaniyah Medical Complex, Manama, Bahrain; 5 Neurosciences Research Center, Tabriz University of Medical Sciences, Tabriz, Iran; 6 Neurology Department, Sa’ad Al-Witry Neurosciences Hospital, Baghdad, Iraq; 7 Neurology Department, Basma Teaching Hospital, Irbid, Jordan; 8 Neurology Department, Jaber AlAhmad Hospital, Kuwait, Kuwait; 9 Department of Medicine, Benghazi Medical Centre, Benghazi, Libya; 10 Neurology Department, Hassan II University Hospital, Sidi Mohamed Ben Abdellah University, Fez, Morocco; 11 Central Stroke Unit, Neuroscience Directorate, Khoula Hospital, Muscat, Oman; 12 Neuroscience Institute, Hamad Medical Corporation, Doha, Qatar; 13 Neurology Department, King Salman ibn Abdulaziz Medical City, Madinah, Saudi Arabia; 14 Neurology Department, Faculty of Medicine and Soba University Hospital, University of Khartoum, Khartoum, Sudan; 15 Neurology and Neurophysiology Department, Damascus Hospital, Damascus, Syria; 16 Department of Neurology, Habib Bourguiba University Hospital, Sfax, Tunisia; Stanford University School of Medicine, UNITED STATES

## Abstract

**Objectives:**

Stroke represents a health care challenge to most parts of the world including the Middle East and North Africa (MENA) region. The MENA represents 6% of the world population with an age-standardized stroke rate of 87.7 (78.2–97.6) per 100,000 population. This number is subject to increase given that the cause of morbidity has recently shifted from infectious diseases to non-communicable diseases. Thus, in the coming years, treatment of stroke will pose a major burden on MENA countries which mostly lie in the low to middle income economies. Accordingly, we need to study the state of MENA stroke services in order to recognize and further inform policy makers about any gaps that need to be bridged in this domain.

**Methods and results:**

Stroke specialists representing 16 countries filled an online survey that included: screening for risk factors, acute management, diagnostics, medications, post-discharge services, and stroke registries. Results showed that 11 countries screen for risk factors, 16 have neuroimaging studies, 15 provide intravenous thrombolysis (IVT), 13 mechanical thrombectomy (MT) while medications for secondary prevention are available in all countries. However, stroke units are not equally available and even absent in 4 countries, and despite the availability of IVT yet, the rate of administration is still low in 6 countries (<5%), and ranges from 5–20% in 7 countries. Stroke registries and training still need to be implemented in most countries.

**Conclusion:**

Although imaging, revascularization therapies and medications for secondary prevention are available in most MENA countries, yet the rate of revascularization is low, so is the number of stroke units insufficient in some countries. Additionally, registries and structured training are still defective. Further field studies are required for more accurate determination of the status of stroke services in the MENA region.

## Introduction

The Middle East and North Africa (MENA) countries account for nearly 6% of the world population [1]. According to the World Bank report of 2022, the MENA region is rich in natural resources yet, most of the countries (14 countries) lie within the low income (LIC), low-middle income (LMIC) and upper-middle income countries (UMIC) [1].

However, it is worth noting that the development in health status in many MENA countries has exceeded the modest economic growth of the region. This was reflected on decreased morbidity and mortality due to achievements in health services [2]. In this context, stroke comes as one of the health challenges that can have a considerable impact on health budgets of MENA countries.

In 2019 nearly 7.3 million stroke cases were reported across the MENA region, with an age-standardized point prevalence ranging from 1136.4 to 2225.7 cases per 100,000 [3]. Thus the direct and indirect effects of stroke continue to influence the states’ health budgets in different countries of the region. On the other hand, stroke is a disorder that can be largely controlled either by primary prevention or by implementation of proper acute management protocols. Hence the emergence of the Middle East and North Africa Stroke Organization (MENASO) that was founded in 2017, aiming “to facilitate communication and create synergies among clinicians and scientists to promote and enhance research and improve clinical outcomes in Stroke” [4]. Accordingly, this study has been fulfilled in collaboration with MENASO.

Although previous research has provided pertinent information about stroke services, yet some studies referred to stroke services in the context of global health systems in the MENA region, others included MENA countries among other low and middle-income countries (LMIC), while some researchers were more concerned about the burden of stroke without referring to available services. Since MENA stroke services were not specifically highlighted. Therefore, the current study is basically focused on the present status of stroke services MENA countries. We aim to identify specific shortcomings that could be presented to health policy makers in order to bridge any gaps in stroke services. Proper management of vascular risk factors and provision of timely acute stroke management can have a positive impact on the economies of the MENA countries.

We have included data from 16 countries of the MENA region provided through a survey that was sent to representative neurologists who are members of MENASO. Details of the survey will be described in the methods and in supplementary files. The results will display the data of different stroke services available in each country namely primary stroke prevention, acute therapy, secondary prevention and post-discharge services. These available services will be compared with those provided in other countries.

## Methods

This cross-sectional observational study was conducted through a comprehensive survey on google forms conveyed to participants via email. We could reach out to representatives of 16 countries all of whom agreed to participate in this survey. All of the participants are members of the MENASO.

The representatives from each of the participating countries were contacted after consensus by the MENASO board. This was based on being active members within their countries in the field of stroke care and management and based upon their active contribution within the MENASO committees. This mainly included participants from the Gulf region as well as Libya, Syria and Sudan. Other Levant representatives (Iraq and Jordan) were contacted through the Angels program based on being active participants in the Angels initiatives (the Angels Initiative is a unique healthcare initiative that helps hospitals around the world become ‘stroke-ready’ so that patients who have just suffered a stroke can be treated as quickly and effectively as possible) and in stroke management. As regards other North African countries, we had previous contact with them in a past study about Stroke services in Africa [5]. The survey design is based upon a similar one performed in a previous study, with some additional questions concerning the availability of certified stroke units/centers, number of stroke units per country, and availability of tenecteplase and/or alteplase as thrombolytic agents.

The survey comprised different sections scanning various stroke services. Among these sections were primary preventive measures available in each country specifically screening for vascular risk factors. Also, the survey included acute stroke services, radiological and laboratory investigations as well as availability of medications used for secondary prevention of stroke. Post discharge services as dedicated stroke rehabilitation, speech therapy, and neuroplasticity modulating services were included as well. Finally, a section was included for availability of continuous medical education for stroke team members, and availability of registries whether national or international.

A WhatsApp group was initiated for participants to facilitate communication, convey the concept of the survey and make sure that responses represented the entire stroke services for each country. This mode of continuous contact with participants also ensured that all their inquiries concerning the survey were answered. This WhatsApp group helped to verify any ambiguities in responses to the survey and to ensure that all participants responded only to questions they were aware of their answers. We always emphasized to give definite data or else tick “not sure”.

All the responses were received, tabulated and analyzed by one center.

This study did not contain human participants. However, each author obtains an approval to participate in this study from the local ethical committee.

### Patient and public involvement

No patient involved.

## Results

A total of 16 countries participated in the study, these countries are, in alphabetical order, Algeria, Bahrain, Egypt, Iran, Iraq, Jordan, Kuwait, Libya, Morocco, Oman, Qatar, Saudi Arabia, Sudan, Syria, Tunisia, United Arab Emirates.

The estimated annual number of new stroke cases among different countries ranged from 1000 to 250,000. And the number of neurologists per 100 thousand population varied being highest in Egypt (4.3) and lowest in Sudan (0.1), while the numbers could not be estimated in Iraq and Syria (Table 1).

**Table 1:** Primary prevention and acute phase management.

				Primary prevention and acute phase management											
Country	Population	Estimated Annual no. of stroke cases	Number of Neurologists per 100000 population **	Screening for modifiable vascular risk factors *	Smoke cessation program	Code stroke	Stroke service provided through			IVT	MT	Other vascular interventions	Craniectomy	Dysphagia assessment	PT within 48 hours from stroke onset
							general hospitals	stroke units	stroke centers						
**No. of countries/%**				**11/16 68.75%**	**7/16 43.75%**	**6/16 37.5%**	**4/16 25%**	**6/16 37.5%**	**6/16 37.5%**	**15/16 93.8%**	**13/16 81.2%**	**15/16 93.75%**	**14/16 87.5%**	**13/16 81.3%**	**14/16 78.5%**
**SA**	34.813.87	18000–25000	1.2	yes	yes	yes	yes	yes	yes	yes	yes	yes	yes	no	no
**UAE**	9.890.402	12000	2.02	yes	yes	yes	yes	yes	yes	yes	yes	yes	yes	yes	yes
**Oman**	5.106.626	2000	0.6	no	no	no	yes	yes	yes	yes	yes	yes	yes	yes	yes
**Iran**	83.992.94	150000	2.02	yes	no	yes	no	yes	yes	yes	yes	yes	yes	yes	yes
**Qatar**	2.881.053	2700	1.2	yes	yes	yes	no	yes	yes	yes	yes	yes	yes	yes	yes
**Bahrain**	1.701.575	1000	1.06	yes	yes	yes	no	yes	yes	yes	yes	yes	yes	yes	yes
**Kuwait**	4.270.571	-	1.05	yes	yes	no	yes	yes	no	yes	yes	yes	yes	no	yes
**Jordan**	10.203.13	-	0.83	yes	no	no	No	yes	yes	yes	yes	yes	yes	yes	yes
**Syria**	17.500.65	30000	-	no	no	no	yes	no	No	no	no	yes	no	yes	yes
**Iraq**	40.222.49	10000–20000	-	no	yes	no	yes	no	no	yes	yes	no	yes	yes	yes
**Libya**	6.871.292	8500	0.58	no	no	no	yes	no	no	yes	no	yes	no	yes	no
**Tunisia**	11.949.32	5000	1.34	yes	no	no	no	yes	yes	yes	no	yes	yes	no	yes
**Morocco**	37.372.80	34000	0.54	yes	yes	yes	yes	yes	yes	yes	yes	yes	yes	yes	yes
**Algeria**	44.686.45	50000	1.34	yes	no	no	no	yes	yes	yes	yes	yes	yes	yes	yes
**Egypt**	104.370.3	200000	4.31	yes	no	no	yes	yes	Yes	yes	yes	yes	yes	yes	yes
**Sudan**	43.849.26	250000	0.11	no	no	no	yes	no	no	yes	yes	yes	yes	yes	yes

*Modifiable vascular risk factors: DM, HTN, Dyslipidemia, Cardiac Diseases, Smoking

**estimated number

SA: Saudi Arabia, UAE: United Arab Emirates, IVT: intravenous thrombolysis, MT: mechanical thrombectomy, PT: physiotherapy

### Primary prevention

**Fig 1:**
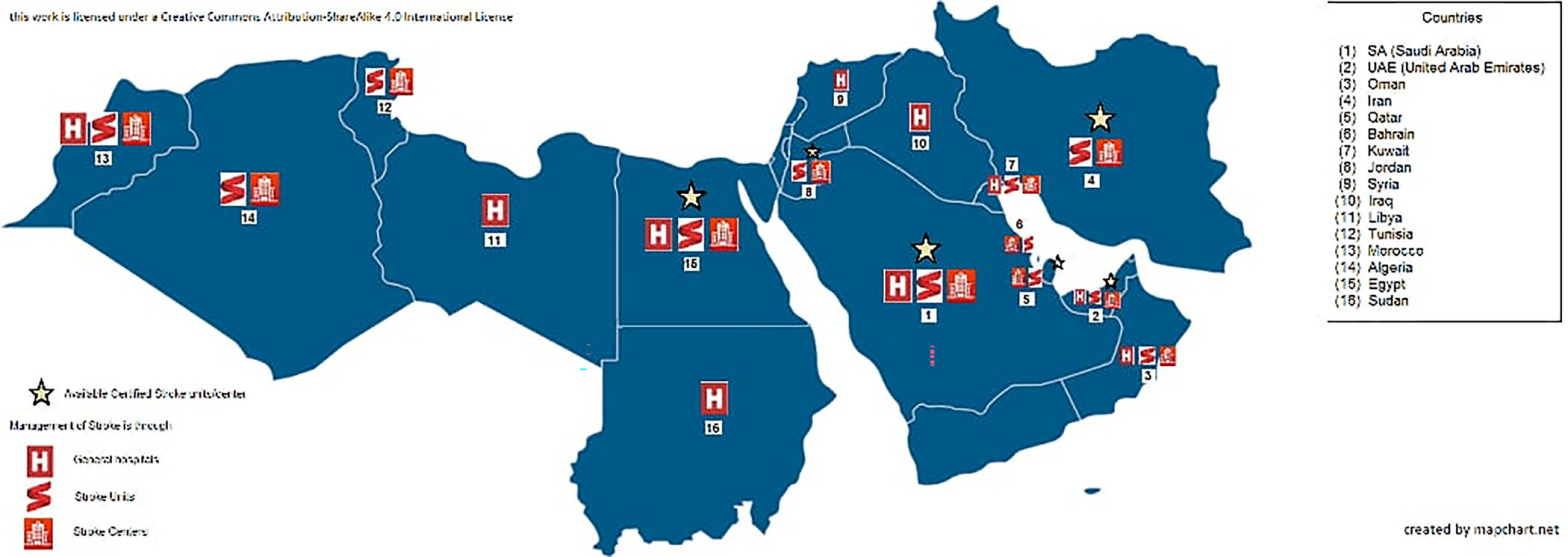
Availability of reperfusion therapies and health facilities providing acute stroke management.

69% of the participating countries reported screening for modifiable stroke risk factors, with variability among factors screened ranging from 37.5% to 69%; diabetes being the highest 69% and smoking the lowest 37.5% (Fig 1, Table 1).

### Acute stroke management: (Table 1)

**Fig 2:**
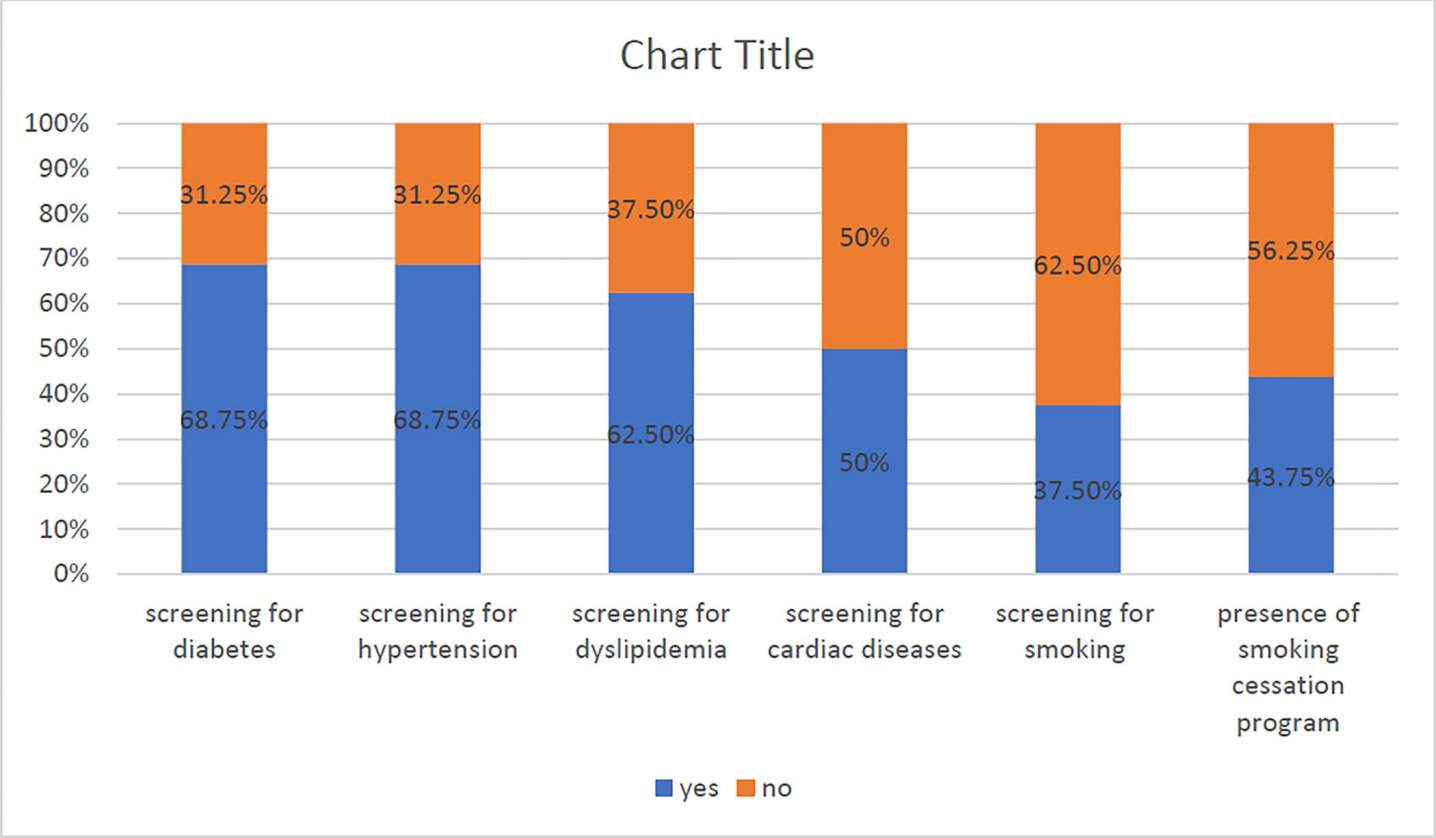
Screening for vascular risk factors.

Stroke units are present in 12 countries, and stroke centers in 11 countries. However, stroke services are exclusive to stroke units or centers in only 6 countries. Stroke service is provided by various bodies in 12 countries; namely stroke ready units, stroke units and centers and general hospitals. While stroke units are totally absent in 4 countries where service is provided by general hospitals (Fig 2).

Code stroke between emergency medical services (EMS) and hospitals is active in 6 of 16 countries, and code stroke within hospitals is present in 6 countries.

Thrombolysis is available and endorsed totally or partially in 15 countries. However, the percentage of cases receiving thrombolytic therapy ranged from <5% in 6 countries to 10–20% in 3 countries. Most countries (12) use Alteplase, and 3 use both Alteplase and Tenecteplase.

As for thrombectomy, it is available in 13 countries, being totally endorsed in 7 and partially endorsed in 5. And despite available in 81% of countries yet the percentage of patients receiving thrombectomy is <5%. Other therapeutic catheters are also available in 15 countries.

Assessment of swallowing is routinely done in the acute setting in 13 countries, and physiotherapy is initiated within 48 hours of admission in 14 countries.

### Diagnostics for acute stroke: (Table 2)

**Table 2:** Secondary prevention and diagnostics.

Secondary Prevention diagnostics								
Country	CT	MRI	Perfusion studies	CTA	Sonographic studies	*Echo screening cardiac sources	Screening for collagen / thrombophilia	Screening for glycated hemoglobin
**No. of countries (%)**	16/16 (100%)	15/16 (93.75%)	10/16 (62.5%)	16/16 (100%)	-	-	14/16 (87.5%)	14/16 (87.5%)
**SA**	yes	yes	yes	yes	CD	transthoracic and transesophageal	no	yes
**UAE**	yes	yes	yes	yes	CD	transthoracic and transesophageal	yes	yes
**Oman**	yes	yes	yes	yes	CD	transthoracic and transesophageal	yes	yes
**Iran**	yes	yes	no	yes	CD and TCD	transthoracic and transesophageal	yes	yes
**Qatar**	yes	yes	yes	yes	CD	transthoracic and transesophageal	yes	yes
**Bahrain**	yes	yes	yes	yes	CD	transthoracic and transesophageal	yes	no
**Kuwait**	yes	yes	yes	yes	not sure	transthoracic and transesophageal	yes	yes
**Jordan**	yes	yes	no	yes	CD	Transthoracic	yes	yes
**Syria**	yes	yes	no	yes	CD	transthoracic and transesophageal	yes	yes
**Libya**	yes	yes	no	yes	none	transthoracic	no	yes
**Tunisia**	yes	yes	yes	yes	CD	transthoracic and transesophageal	yes	yes
**Morocco**	yes	no	no	yes	CD	transthoracic	yes	yes
**Algeria**	yes	yes	yes	yes	CD	transthoracic and transesophageal	yes	yes
**Egypt**	yes	yes	yes	yes	CD and TCD	transthoracic and transesophageal	yes	yes
**Iraq**	yes	yes	no	yes	CD	transthoracic and transesophageal	yes	no
**Sudan**	yes	yes	yes	yes	CD	transthoracic	yes	yes

SA: Saudi Arabia; UAE: United Arab Emirates, CT: Computed tomography, MRI: Magnetic resonance imaging, CTA: Computed Tomography Angiography, CD: carotid duplex, TCD: transcranial Doppler.

*Echo screening: transesophageal or transthoracic

#### Radiologic studies

CT and CT angiography are performed in all countries, MRI stroke protocol in 15 countries, MRA and MRV (Magnetic resonance venography) in 13, diagnostic angiography in 13, perfusion studies in 10 and MRI vessel wall imaging in 7.

#### Sonographic studies

Carotid duplex is conducted in 12, while carotid duplex and transcranial doppler in 2 countries. Investigation for cardio embolic sources is done by echo-cardiography in 4 countries.

In-hospital investigations for long term control of diabetes by glycosylated hemoglobin, collagen diseases, thrombophilia and vasculitis for stroke in young are available in 14 countries (Table 2).

### Available medications for management of acute ischemic stroke: (Table 3)

**Table 3:** Available medications for primary and secondary stroke prevention.

Variables	Frequency	Percentage
Thrombolysis	15/16	93.8%
Endorsement of thrombolysis		
Total	10/15	66.7%
Partial	5/15	33.3%
*Percentage of thrombolysis to total stroke cases*		
None	1/15	6.7%
<5%	6/15	40%
5–10%	4/15	26.7%
10–20%	3/15	20%
Not sure	1/15	6.7%
Type of thrombolytic agent in use		
Alteplase	15/16	93.75%
Tenecteplase	3/16	18.75%
Both	3/16	18.75%
Neither	1/16	6.25%
*Average door to needle time in hours*		
Not applicable (thrombolytic available yet not administered)	1/15	6.7%
Less than half an hour	0/15	0%
1–2 hours	3/15	20%
2–3 hours	1/15	6.7%
3–4 hours	1/15	6.7%
not sure	9/15	60%
Thrombectomy	13/16	81.25%
Endorsement of thrombectomy		
Total	7/13	53.8%
Partial	5/13	38.5%
Not endorsed	1/13	7.7%
Percentage of thrombectomy to total stroke cases		
<5%	7/16	43.75%
Not sure	6/16	37.5%
Therapeutic catheters / coiling / stents for aneurysms	15/16	93.8%
Endorsement of the therapeutic catheters		
Total	6/15	40%
Partial	7/15	46.7%
Not endorsed	2/15	13.3%
Aspirin (Acetyl Salicylic Acid)	16/16	100%
Clopidogrel	16/16	100%
Prasugrel	4/16	25%
Ticagrelor	7/16	43.8%
Cilostazol	3/16	18.8%
Lipid lowering agents (Statins)	16/16	100%
Diuretics	16/16	100%
BB	16/16	100%
ACEI	16/16	100%
ARBS	16/16	100%
CCB	16/16	100%
Alpha Blockers	14/16	87.5%
Alpha 2 receptor antagonists	10/16	62.5%
Central Agonists	13/16	81.2%
Peripheral Adrenergic Inhibitors	12/16	75%
Vasodilators	15/16	93.7%
Insulin and oral hypoglycemics in their different forms	16/16	100%
Warfarin	16/16	100%
Rivaroxaban	16/16	100%
Dabigatran	13/16	81.2%
Apixaban	15/16	93.7%
Edoxaban	4/16	25%
Heparin and low Molecular Weight Heparin	16/16	100%
Multi-modal neuropeptide medication (cerebrolysin)	5/16	31.3%
Different brain dehydrating measures	16/16	100%
Dedicated rehabilitation program post discharge	11/16	68.75%
Neuroplasticity enhancing modalities (trans-cranial magnetic stimulation, direct current stimulation)	3/16	18.8%
Stroke awareness campaigns	10/16	62.5%

BB: Beta Blockers, ACEI: Angiotensin converting enzyme inhibitors, ARBS: Angiotensin II receptors blockers, CCB: Calcium Channel blockers

Aspirin and clopidogrel are available in all countries, Ticagrelor in 7, Prasugrel in 4 and Cilostazol in 3. Lipid-lowering agents and anti-hypertensives (diuretics, beta blockers, ACE inhibitors, Angiotensin II receptor blockers, calcium channel blockers) are present in all countries, with variable availability of other antihypertensive agents.

Medications for treatment of diabetes whether insulin or oral hypoglycemics are available in all countries. Some form of injectable and oral anticoagulants, whether warfarin or novel oral anticoagulants show variable availability in different countries, with at least one form being available in each country (Table 3).

### Post-discharge services: (Table 4)

**Table 4:** Post discharge and quality management.

Post Discharge and Quality Management								
Country		Dedicated Rehabilitation program	Neuroplasticity enhancing modalities	Dedicated Speech therapy	Psychiatric consultation and social support	National stroke registry system	International stroke registry database	Stroke awareness campaign
**No. of countries (%)**		11/16 (68.75%)	3/16 (18.8%)	8/16 (50%)	8/16 (50%)	5/16 (31.3%)	8/16 (50%)	10/16 (62.5%)
**Endorsement**	**total**	4/16 (25%) 6/16 (37.5%)	- -	5/16 (31.25%) 6/16 (37.5%)	- -	- -	- -	- -
	**partial**							
**SA**		yes	no	yes	yes	yes	no	yes
**UAE**		yes	yes	yes	yes	yes	yes	yes
**Oman**		yes	no	yes	yes	no	no	yes
**Iran**		no	yes	no	yes	yes	yes	yes
**Qatar**		yes	no	yes	yes	no	yes	yes
**Bahrain**		no	no	no	no	no	yes	no
**Kuwait**		yes	no	yes	no	no	no	no
**Jordan**		yes	no	yes	yes	yes	yes	yes
**Syria**		no	no	no	no	no	no	yes
**Libya**		yes	no	no	no	no	no	no
**Tunisia**		no	no	no	no	no	no	no
**Morocco**		yes	no	yes	yes	no	yes	yes
**Algeria**		yes	no	yes	no	no	yes	yes
**Egypt**		no	yes	no	no	yes	yes	no
**Iraq**		yes	no	no	no	no	no	no
**Sudan**		yes	no	no	yes	no	no	yes

SA: Saudi Arabia; UAE: United Arab Emirates

Stroke rehabilitation protocols are provided in 11 countries while neuroplasticity enhancing modalities as non-invasive brain stimulation are present only in 3 countries. Speech therapy as well as psychiatric services or social support after discharge are offered in 8 regions. (Table 4)

### Registration and training protocols

International stroke data base registries are implemented in 8 regions and national registries in 5. Stroke management training for doctors and nurses is supplied in 8 countries and campaigns for nationwide awareness about stroke are running in 10 countries. (Table 4)

## Discussion

This study represents most countries of the MENA region as it includes representatives from North Africa, Gulf and Levant regions. The results revealed disparity in stroke service among various countries. Screening for risk factors across MENA region reached 69%. A study of stroke services in 84 countries across the globe showed that one third of primary preventive activities were fulfilled by the studied hospitals, and that MENA countries responded positively to 44% of questions about stroke prevention [6].

In our study, most of the countries screened for diabetes. This coincides with the WHO global agreement to reduce the rise in diabetes by 2025 [7]. As for smoking, despite being a major risk for stroke yet smoking cessation programs are present in a few countries. This is similar to many African countries nevertheless, other developing countries achieved reduction of tobacco use through implementing comprehensive antismoking programs. Similarly, Mauritius, the Former Soviet Union, Japan and Taiwan were successful in that aspect through mass health promotion.

The number of stroke units showed marked diversity among MENA countries. The Gulf region had the highest number of stroke units followed by North Africa, with the least number in Levant region, while 4 countries had no stroke units. This can be explained by the variability of socioeconomic levels among those regions. Several stroke units are certified by different bodies [8] and it is worth mentioning that some countries as Saudi Arabia had no stroke units in 2011 [9], but by 2022 reported the presence of certified stroke units.

In other developing countries, as Brazil, despite having 156 centers yet it suffers a marked decline in quality of stroke services in general hospitals compared to specialized stroke centers [10]. Other Latin American countries have a few stroke centers or even do not monitor stroke services [6]. In contrast, 95% of European countries have stroke units [11] compared to only 75% of MENA countries.

IVT is available and endorsed in most MENA countries. This is comparable to HICs that showed 60% availability of IVT, in contradistinction to 26% in LICs. Nevertheless, the rate of thrombolysis is still low in most MENA countries. Some Latin American countries reported <1% IVT, [10] while the rate ranged from 7% to 23.5% in various parts of Europe [9, 12].

Concerning door to needle time, nearly a quarter of the countries reported less than 2 hours-time mostly due to the convenience of CT scan in practically all surveyed countries. Failure to administer IVT has been previously attributed to lack of trained personnel [11] or radiological evaluation in some countries.

MT is another revascularization therapy that is available in most MENA countries, compared to a recent study that reported MT in 30.5% of countries worldwide [6]. However, MT is performed in less than 5% of cases. Despite that MT is supposed to improve outcome in patients arriving later than the time window for IVT. Yet it is not endorsed in most MENA policies.

Routine screening for dysphagia on admission is performed in most MENA countries. This is much higher than previously reported in nearby African countries (47%) [5]. Screening for dysphagia can guard against aspiration pneumonia and thus shorten the duration of hospital stay [13].

Other services related to acute stroke management as code stroke are not fully established being only available in Morocco, and gulf countries. Implementing Code stroke may be difficult in some countries as Egypt where most stroke cases arrive to hospitals by private cars not by emergency medical services [14]. This point represents a serious gap in healthcare services that should be attended to by the ministries of health due to its impact on onset to door time and consequently on the rate of IVT as well as MT.

Post-discharge services as medications for secondary prevention are mostly available in all MENA regions. At least 2 antiplatelets are available in every country, so are lipid lowering agents, antihypertensives, hypoglycemics, anticoagulants and at least one type of novel oral anticoagulants.

Rehabilitation services are provided in some of the MENA countries yet, they are nearly absent in others. Although early initiation of rehabilitation is associated with shorter institutional stay and less disability [15] yet we noted that only few countries endorse this service. Similar variability in rehabilitation services was reported in Thailand, and in other LMICs the level was 30% less than that recommended by American Heart Association [6]. Other complementary services like speech therapy, neuroplasticity enhancing modalities, psychiatric consultation and social support are present in a few countries. Similar to our findings, these services still lack uniformity in LMICs across Africa and Asia [16].

We could perceive a gap pertaining to stroke registries with only half of the MENA countries adopting registries compared to 59% of European countries [11]. Registries are supposed to improve stroke care through pointing out defects in stroke pathways within hospitals in order to perform corrective actions [17].

Finally, services that indirectly enhance stroke care like embracing stroke awareness campaigns and training programs for healthcare providers are missing in six countries. In comparison, these services were reportedly administered in only 41% of African countries [5, 18].

As for the number of adult neurologists, all countries except one lay below the numbers present in HICs. According to the report of WHO, this number ranges from a median of 0.03 per 100000 population in LICs to 4.75 per 100000 population in HICs [19].

## Conclusion

In this study we investigated the availability of stroke services in the MENA region. We could find appreciable efforts in the domain of primary prevention, yet still more serious actions are needed to combat smoking. The availability and early administration of IVT in hospitals within the window can be attributable to the presence of neuroimaging modalities in most countries. Also other in-hospital services are relatively available as dysphagia screening and rehabilitation. Medications for secondary prevention are attainable in practically all countries.

On the other hand, stroke units need to be equally distributed across the MENA region, with augmentation of the rate of administration of revascularization therapies. Secondary prevention is available in many countries, but still needs to be more widely developed. Governmental support is required for enhancing emergency medical services EMS, securing post-discharge services and implementing stroke training programs.

### Limitations of the study

The data presented in this study are derived from a survey, did not involve all countries and might not comprise all information about stroke services. It included one or two representatives from each country and participants could not necessarily answer all inquiries.

### Future research

Future research based on national registries from all countries is required to provide more pertinent information. Also, further studies are needed to explore the impact of this study on improving stroke services in relevant countries.

## Supporting information

S1 Data (XLSX)

## References

[1] “World Bank Country and Lending Groups.” https://datahelpdesk.worldbank.org/knowledgebase/articles/906519-world-bank-country-and-lending-groups.

[2] Mate K., Bryan C., Deen N., and McCall J., “Review of Health Systems of the Middle East and North Africa Region,” *Int*. *Encycl*. *Public Heal*., no. January, pp. 347–356, 2016, doi: 10.1016/B978-0-12-803678-5.00303–9

[3] Jaberinezhad M. et al., “The burden of stroke and its attributable risk factors in the Middle East and North Africa region, 1990–2019,” *Sci*. *Rep*., vol. 12, no. 1, pp. 1–11, 2022, doi: 10.1038/s41598-022-06418-x 35177688 PMC8854638

[4] “MENA STROKE.” http://menastroke.org/.

[5] Roushdy T. et al., “Stroke services in Africa: What is there and what is needed,” *Int*. *J*. *Stroke*, vol. 00, no. X, 2022, doi: 10.1177/17474930211066416 35034522

[6] Owolabi M. O. et al., “The state of stroke services across the globe: Report of World Stroke Organization–World Health Organization surveys,” *Int*. *J*. *Stroke*, vol. 16, no. 8, pp. 889–901, 2021, doi: 10.1177/17474930211019568 33988062 PMC8800855

[7] “WHO. Diabetes.” https://www.who.int/health-topics/diabetes#tab=tab_1.

[8] Aref H., Zakaria M., Shokri H., Roushdy T., El Basiouny A., and El Nahas N., “Changing the Landscape of Stroke in Egypt,” *Cerebrovasc*. *Dis*. *Extra*, vol. 11, no. 3, pp. 155–159, 2021, doi: 10.1159/000521271 34864736 PMC8787609

[9] Al Khathaami A. M., Algahtani H., Alwabel A., Alosherey N., Kojan S., and Aljumah M., “The status of acute stroke care in Saudi Arabia: An urgent call for action!,” *Int*. *J*. *Stroke*, vol. 6, no. 1, pp. 75–76, 2011, doi: 10.1111/j.1747-4949.2010.00542.x 21205244

[10] Ouriques Martins S. C. et al., “Priorities to reduce the burden of stroke in Latin American countries,” *Lancet Neurol*., vol. 18, no. 7, pp. 674–683, 2019, doi: 10.1016/S1474-4422(19)30068-7 31029579

[11] Aguiar de Sousa D. et al., “Access to and delivery of acute ischaemic stroke treatments: A survey of national scientific societies and stroke experts in 44 European countries,” *Eur*. *Stroke J*., vol. 4, no. 1, pp. 13–28, 2019, doi: 10.1177/2396987318786023 31165091 PMC6533860

[12] Mikulik R. et al., “Stroke 20 20: Implementation goals for intravenous thrombolysis,” *Eur*. *Stroke J*., vol. 6, no. 2, pp. 151–159, 2021, doi: 10.1177/23969873211007684 34414290 PMC8370063

[13] Vose A., Nonnenmacher J., Singer M. L., and González-Fernández M., “Dysphagia Management in Acute and Sub-acute Stroke,” *Curr*. *Phys*. *Med*. *Rehabil*. *Reports*, vol. 2, no. 4, pp. 197–206, 2014, doi: 10.1007/s40141-014-0061-2 26484001 PMC4608439

[14] Aref H. M., Shokri H., Roushdy T. M., Fathalla, and El Nahas N. M., “Pre-hospital causes for delayed arrival in acute ischemic stroke before and during the COVID-19 pandemic: A study at two stroke centers in Egypt,” *PLoS One*, vol. 16, no. 7 July, pp. 1–11, 2021, doi: 10.1371/journal.pone.0254228 34260632 PMC8279320

[15] Clarke D. J., “The role of multidisciplinary team care in stroke rehabilitation,” *Prog*. *Neurol*. *Psychiatry*, vol. 17, no. 4, pp. 5–8, 2013, doi: 10.1002/pnp.288

[16] Chimatiro G. L. and Rhoda A. J., “Scoping review of acute stroke care management and rehabilitation in low and middle-income countries,” *BMC Health Serv*. *Res*., vol. 19, no. 1, 2019, doi: 10.1186/s12913-019-4654-4 31684935 PMC6829977

[17] Asplund K. et al., “The Riks-Stroke story: Building a sustainable national register for quality assessment of stroke care,” *Int*. *J*. *Stroke*, vol. 6, no. 2, pp. 99–108, 2011, doi: 10.1111/j.1747-4949.2010.00557.x 21371269

[18] Wyber R., Vaillancourt S., Perry W., Mannava P., Folaranmi T., and Celi L. A., “Big data in global health: improving health in low- and middle-income countries,” *Bull*. *World Health Organ*., vol. 93, no. 3, pp. 203–208, 2015, doi: 10.2471/BLT.14.139022 25767300 PMC4339829

[19] “WHO and the World Federation of Neurology. Atlas: country resources for neurological disorders, 2nd ed.,” [Online]. Available: https://www.who.int/publications/i/item/9789241565509.

